# miR-Novel-80 Suppresses Porcine Reproductive and Respiratory Syndrome Virus Replication by Targeting the Viral *Nsp1* Gene and Downregulating Host CXXC Finger Protein 4

**DOI:** 10.3390/ani16152434

**Published:** 2026-08-06

**Authors:** Shuo Feng, Yiwen Pei, Xue Gao, Danjiao Yang, Jie Liu, Zijing Guo, Zhidong Zhang, Long Zhou

**Affiliations:** 1 Key Laboratory of Animal Medicine of Sichuan Education Department, College of Animal Science and Veterinary Medicine, Southwest Minzu University, Chengdu 610041, China; fengshuo0418@163.com (S.F.); 19981253316@163.com (Y.P.); gaoxue3201@163.com (X.G.); jieliuhzau@163.com (J.L.); guozijing@swun.edu.cn (Z.G.); zhangzhidong@swun.edu.cn (Z.Z.); 2 Institute of Animal Science of Ganzi Tibetan Autonomous Prefecture of Sichuan Province, Kangding 626000, China; kangyang2468@126.com; 3 Key Laboratory of Ministry of Education and Sichuan Province for Qinghai-Tibetan Plateau Animal Genetic Resource Reservation and Utilization, Chengdu 610041, China

**Keywords:** porcine reproductive and respiratory syndrome virus (PRRSV), microRNA, CXXC finger protein 4 (*CXXC4*), nsp1

## Abstract

Porcine reproductive and respiratory syndrome (PRRS), caused by porcine reproductive and respiratory syndrome virus (PRRSV), is a major infectious disease that poses a severe threat to the global swine industry. To investigate the role of miRNAs in the infection and susceptibility of PRRSV, four miRNA libraries were constructed and sequenced from PRRSV-infected and mock-infected of Tibetan pigs and Large White pigs at 7 days post-infection. A novel miRNA, miR-novel-80, was differentially expressed between PRRSV-infected and mock-infected porcine alveolar macrophages from 2 pig breeds. Importantly, the over-expression of miR-novel-80 inhibited the replication of a PRRSV-1 strain and multiple lineages (L1, L5, and L8) of PRRSV-2 strains in a dose-dependent manner. Bioinformatic predictions and experimental validation demonstrated that miR-novel-80 restricts viral replication through a dual antiviral mechanism. Directly, it targets the PRRSV *nsp1*-coding region within the ORF1a to suppress viral proliferation. Indirectly, miR-novel-80 specifically down-regulates the expression of host factor CXXC finger protein 4 (*CXXC4*). This reduction relieves the suppression of the Wnt/β-catenin signaling pathway, which in turn activates NF-κB-dependent innate immune responses to further inhibit PRRSV infection. Collectively, this study investigates the biological characteristics of miR-novel-80 and unveils its underlying molecular mechanisms in restricting PRRSV infection in vitro. However, its biological function and anti-PRRSV therapeutic effect in vivo need further investigation.

## Simple Summary

Host microRNAs can affect RNA virus replication and pathogenesis through direct binding to the viral genome or via transcriptome-level changes triggered by the virus. In the study, we investigated the role of a novel miRNA (miR-novel-80) in regulating the viral replication of porcine reproductive and respiratory syndrome virus (PRRSV). Over-expression of miR-novel-80 inhibited the replication of a PRRSV-1 strain and multiple lineages of PRRSV-2 isolates in a dose-dependent manner. Mechanistically, the antiviral activity of miR-novel-80 was attributable to the direct targeting of the PRRSV *nsp1* gene and indirect activated the Wnt/β-catenin signaling pathway via inhibiting the expression of host CXXC finger protein 4 (*CXXC4*). In summary, this study identifies miR-novel-80 as a novel regulator of PRRSV replication characterized by a dual mechanism, namely the direct targeting of the viral *nsp1* gene and the indirect activation of the Wnt/β-catenin pathway via host *CXXC4* suppression, providing valuable mechanistic insights into miRNA-mediated antiviral defense against PRRSV.

## 1. Introduction

Porcine reproductive and respiratory syndrome (PRRS), a highly contagious and transmissible disease caused by porcine reproductive and respiratory syndrome virus (PRRSV), results in severe reproductive disorder in sows and respiratory disease in domesticated pigs [1,2]. PRRSV was initially documented in the United States of America in 1987, and outbreaks have since spread throughout North America, Europe, and Asia [3]. According to antigenicity, PRRSV strains can be classified into two distinct species, *Betaarterivirus suid 1* (PRRSV 1) and *Betaarterivirus suid 2* (PRRSV 2) [4,5], each comprising multiple lineages with substantial genetic variation. Currently, PRRS poses a considerable threat to the global swine industry [6,7]. It is estimated that the economic production loss to U.S. swine producers is approximately $1.2 billion annually. In Europe, the loss ranges from €3 to €160 per sow, while in China, the cost per sow from breeding to fattening is approximately Chinese Yuan (CNY) 1424 [8,9].

Given the persistent threat of PRRS, genetic control is considered a viable strategy [10]. The Tibetan pig is a Chinese indigenous pig breed on the Qinghai–Tibet Plateau. Having long inhabited harsh natural environments at an average altitude exceeding 3000 m, this breed exhibits strong stress resistance. A previous study demonstrated that compared with Large White pigs, Tibetan pigs exhibited milder clinical manifestations and significantly lower viremia when they were artificially infected with PRRSV in vivo [11,12]. However, the molecular mechanisms regulating host genes to suppress PRRSV replication remain poorly understood.

MicroRNAs (miRNAs) are evolutionarily conserved small non-coding RNAs [13,14] and play important roles in numerous biological processes, such as cell development, metabolism, apoptosis, and immune responses [15,16]. Multiple studies have shown that host miRNAs can affect PRRSV replication and pathogenesis through direct binding to the viral RNA genome or via transcriptome-level changes triggered by the virus [17]. Notably, some miRNAs have been demonstrated to be potent antiviral agents against PRRSV in vitro and in vivo, such as miR-361-3p, miR-130b, and let-7 [18,19,20], indicating the potential applicability of miRNAs in the clinical treatment of PRRS. In this study, we identified a novel miRNA (miR-novel-80) from PRRSV-infected Tibetan pigs and control Large White pigs using high-throughput sequencing and elucidated that it restricts PRRSV infection through a potent dual antiviral mechanism. Directly, miR-novel-80 targets the PRRSV *nsp1*-coding region within the ORF1a region of the viral genome to suppress viral replication. Indirectly, it targets and downregulates the host factor *CXXC4*, a critical negative regulator of the Wnt/β-catenin signaling pathway [21,22]. Furthermore, we demonstrated that the targeted suppression of *CXXC4* activates the Wnt/β-catenin cascade, which in turn drives NF-κB activation to mount a robust antiviral defense against PRRSV.

## 2. Materials and Methods

### 2.1. Cell Lines and Viruses

MARC-145 and 293T cells were cultured in Dulbecco’s Modified Eagle Medium (DMEM; Servicebio, Wuhan, China) supplemented with 10% fetal bovine serum (FBS; Sperikon, Luzhou, China). Immortalized porcine alveolar macrophage (iPAM) cells were cultured in RPMI 1640 medium (Gibco, Waltham, MA, USA) containing 10% FBS. All cell lines were propagated at 37 °C in a humidified incubator under an atmosphere of 5% CO_2_. All cell lines used in this study were purchased from the Beina Culture Collection (BNCC, Beijing, China). The PRRSV strains used in this study, including the SCwhn14XC-1 (JXA1-like GenBank accession no. KT030989), SC202403 (NADC30-like PV779109), SC202401 (VR2332-like PV779107), and SC202404 (BJEU06-1-like PV533618) strains, were maintained in our laboratory.

### 2.2. Animals

A total of 12 one-month-old healthy weaned piglets (six Tibetan pigs and six Large White pigs) were purchased from a commercial pig farm in the Ganzi Tibetan Autonomous Prefecture of Sichuan Province, China. All animals were confirmed to be negative for PRRSV, Pseudorabies virus (PRV), and Porcine Circovirus (PCV) prior to the experiment using both molecular methods (PCR/RT-PCR) and antibody detection (ELISA). For each breed, piglets were randomly assigned to either the PRRSV infection group (*n* = 3 per breed) or the control group (*n* = 3 per breed). Piglets in the PRRSV challenge group were inoculated intranasally (1 mL) and intramuscularly (1 mL) with PRRSV JXA1-like (TCID_50_ = 10^4.89^/mL). At 7 days, all piglets were humanely euthanized by an intravenous overdose of sodium pentobarbital (100 mg/kg; Sigma-Aldrich, St. Louis, MO, USA). Death was confirmed by the cessation of cardiopulmonary function for a minimum of 5 min, after which porcine alveolar macrophages (PAMs) were harvested via bronchoalveolar lavage (BAL) using phosphate-buffered saline (PBS). Animal experiments in this study were approved by the Animal Ethics Committee of Southwest Minzu University and strictly followed the guidelines of the Institutional Animal Care and Use Committee.

### 2.3. miRNA Sequencing and Histopathological Analysis

At 7 days post-infection, lung tissues were collected from the challenged pigs, and porcine alveolar macrophages (PAMs) were isolated for small RNA sequencing. A total of 12 samples were used to construct cDNA libraries. After filtering against the Rfam, cDNA, and repeat sequence databases to remove non-miRNA components such as tRNA, snRNA, and rRNA, the remaining sequences were aligned to the miRbase 19.0 (http://www.mirbase.org/) database to identify known miRNAs. In addition, tissue samples, including lungs and lymph nodes, were collected. Tissue samples were fixed in 4% paraformaldehyde, embedded in paraffin, sectioned, and stained with H&E for histopathological examination.

### 2.4. Transfection of miRNAs and CXXC4 Overexpression

MicroRNA (miRNA) mimics for porcine miR-novel-686, miR-novel-79, miR-novel-13, miR-novel-80, and miR-novel-970, as well as a miR-novel-80 inhibitor, were designed and synthesized by Tsingke Biotechnology (Beijing, China). MARC-145 or iPAM cells were seeded at a density of 5 × 10^5^ cells per well in 6-well plates. When the cells reached 70–80% confluence, they were transfected with miRNAs at a final concentration of 60 nM. At 24 h post-transfection, cells were infected with the PRRSV JXA1-like strain at an MOI of 0.05. Small interfering RNAs (siRNAs) targeting porcine *CXXC4* were designed and synthesized by GeneCreate (Wuhan, China). The sequences of all miRNA mimics, inhibitors, and siRNAs used in this study are detailed in Table 1. Cell transfections were performed using Lipofectamine 2000 (Invitrogen, Carlsbad, CA, USA) according to the manufacturer’s instructions. At the designated time points post-transfection, cells were inoculated with PRRSV at a multiplicity of infection (MOI) of 0.1 or mock-infected with control medium for 2 h. Following viral adsorption, the inoculum was replaced with maintenance medium containing 2% FBS, and the cells were incubated for an additional 24 h before being harvested for Western blotting (WB) or quantitative RT-qPCR analysis.

### 2.5. Western Blotting Analysis

MARC-145 cells or iPAMs were lysed in RIPA buffer containing 1% phenylmethylsulfonyl fluoride (PMSF). The proteins were separated on 12% SDS-PAGE and transferred to polyvinylidene fluoride (PVDF) membranes. The following primary antibodies were used for WB: anti-PRRSV N protein (GeneTex, Irvine, CA, USA; 1:2000), anti-GAPDH (Affinity, San Francisco, CA, USA; 1:2000), anti-CXXC4 (Proteintech, Wuhan, China; 1:2500), and anti-β-catenin (HuaBio, Hangzhou, China; 1:2000). Anti-rabbit IgG (Invitrogen, Carlsbad, CA, USA; 1:5000) was used as the secondary antibody. Normalization was performed using GAPDH as a loading control, and the results were quantified using the ImageJ 1.54g software.

### 2.6. RNA Extraction and Quantitative PCR

For RNA extraction, total RNA was extracted from MARC-145 cells or iPAMs using the TRIzol method (Life-iLab, Shanghai, China). The RNA was then reverse transcribed into cDNA using the EXONGEN reverse transcription kit (EXONGEN, Chengdu, China). qRT-PC-R was performed to determine mRNA levels. The relative mRNA expression was calculated using the 2^−ΔΔCT^ method, with GAPDH and β-actin as internal controls. PRRSV copy numbers were determined by absolute quantification using a standard curve. The primers used for qRT-PCR are listed in Table 2.

### 2.7. Dual Luciferase Reporter Gene Assay

The 3′UTR of *CXXC4* or the ORF1a fragment containing the putative miR-novel-80 binding site (WT), as well as the corresponding sequence harboring the mutated binding site (MUT), was cloned into the pmirGLO vector (GeneCreate, Wuhan, China), generating pmirGLO-CXXC4-WT, pmirGLO-CXXC4-MUT, pmirGLO-ORF1a-WT, and pmirGLO-ORF1a-MUT. 293T cells were co-transfected with pmirGLO-CXXC4-WT, pmirGLO-CXXC4-MUT, pmirGLO-ORF1a-WT, or pmirGLO-ORF1a-MUT plasmids, together with either miR-novel-80 mimics, miR-novel-80 inhibitor, or negative control, using Lipofectamine 2000 (Invitrogen, Cashman, CA, USA). After 24 h, firefly and Renilla luciferase activities were measured sequentially using the dual-luciferase assay kit (Vazyme, Nanjing, China), and the relative luciferase activity was calculated.

### 2.8. Transcriptome Sequencing and Data Analysis

iPAMs received transfected with miR-novel-80 or miR-NC for 24 h, with three biological replicates performed per group. Total RNA was then extracted using TRIzol reagent. Transcriptome sequencing and subsequent data analysis were performed by OE Biotech, Inc. (Shanghai, China).

### 2.9. miRNA Target Prediction

The genes sequences of the CXXC4 3′UTR and the PRRSV genomes from different lineages were obtained from NCBI (https://www.ncbi.nlm.nih.gov/, accessed on 3 August 2026). The online tool miRanda (v3.3a) was used to predict the targets of miR-novel-80.

### 2.10. Statistical Analysis

All experiments were independently repeated at least three times. Statistical analyses were performed using GraphPad Prism v10.1.2. Gene interaction analysis and visualization were conducted using STRING (https://string-db.org/) and Cytoscape v3.10.4. Using Figdraw (v2.0, https://www.figdraw.com/) to create figures. Differences between groups were analyzed using Student’s *t* test or two-way analysis of variance (two-way ANOVA), as appropriate. Significance levels are indicated in the figures as follows: * *p* < 0.05, ** *p* < 0.01, *** *p* < 0.001, **** *p* < 0.0001; ns, not significant.

## 3. Results

### 3.1. miR-Novel-80 Inhibits PRRSV Replication

**Figure 1:**
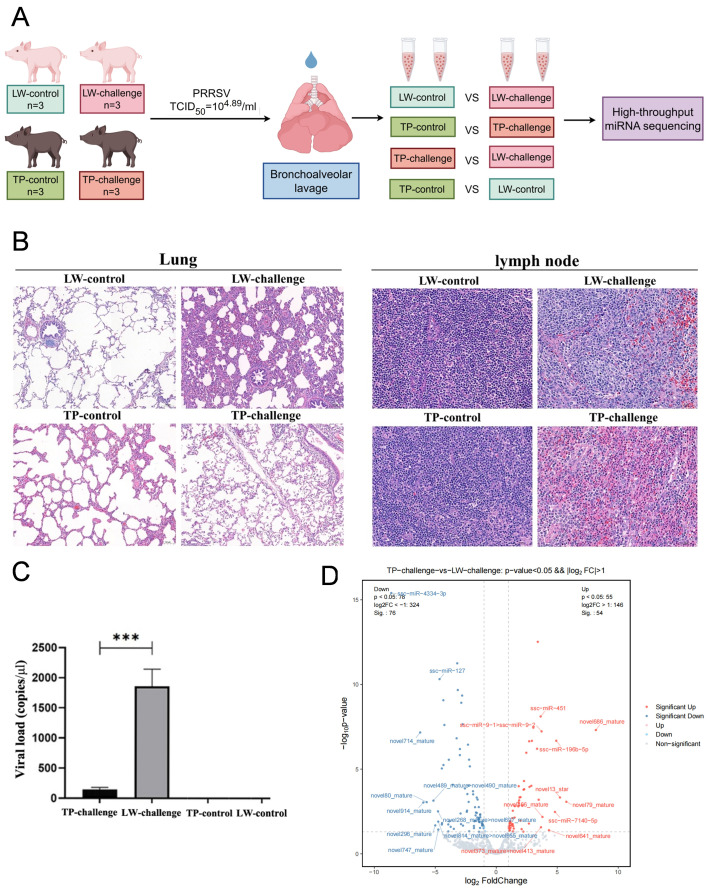
miRNA screening and histopathological analysis in Tibetan and Large White pigs. (**A**) Schematic overview of the microRNA sequencing workflow in Tibetan and Large White pigs. (**B**) Histopathological examination of lung and lymph node tissues from Tibetan and Large White pigs following PRRSV infection. (**C**) RT-qPCR analysis of PRRSV RNA levels in lung tissues from TP-challenged, LW-challenged, TP-control, and LW-control pigs. *** *p* < 0.001. (**D**) Volcano plot of differentially expressed miRNAs in TP challenge vs. LW challenge.

In this study, twelve 4-week-old piglets, comprising six Tibetan pigs (TP) and six Large White pigs (LW), were used. PAMs were collected at 7 dpi for miRNA sequencing and analysis. The microRNA sequencing data was aligned against multiple databases (Figure 1A). Histopathological examinations showed that LW-challenged pigs exhibited mild to moderate interstitial pneumonia, accompanied by prominent pulmonary emphysema and marked pulmonary congestion. TP-challenged pigs developed focal interstitial pneumonia, accompanied by extensive compensatory pulmonary emphysema. The lymph nodes in the LW-challenged group displayed infiltration of eosinophils and neutrophils with multifocal necrosis. The lymph nodes in the TP-challenged group showed moderate neutrophilic infiltration and mild lymphocytic degeneration and necrosis. In contrast, no significant pathological changes were observed in either the TP-control or LW-control groups (Figure 1B). To further confirm PRRSV infection in the challenged animals, we measured viral RNA levels in lung tissues by RT-qPCR. The viral load was significantly higher in the LW-challenged group compared to the TP-challenged group, whereas no viral RNA was detected in either the TP-control or LW-control groups (Figure 1C). Unaligned sequences that did not match the miRBase database were subjected to novel miRNA prediction. Differential expression analysis revealed a total of 355 known miRNAs and 1150 novel miRNAs. Among these, 81 miRNAs were identified in the Large White infection group versus the Large White control group (LW challenge vs. LW control), 22 in the Tibetan infection group versus the Tibetan control group (TP challenge vs. TP control), 130 in the Tibetan infection group versus the Large White infection group (TP challenge vs. LW challenge), and 175 in the Tibetan control group versus the Large White control group (TP control vs. LW control). Differentially expressed miRNAs between the Tibetan infection group and the Large White infection group (TP challenge vs. LW challenge) are presented in the volcano plot (Figure 1D). Interestingly, the number of novel miRNAs was greater than that of known miRNAs. Accumulating evidence has demonstrated that known miRNAs play a critical role in modulating viral replication. Specifically, miR-204 restricts PRRSV replication by inhibiting LC3B-mediated autophagy [23], whereas miR-24-3p promotes viral replication by downregulating heme oxygenase-1 (HO-1) expression [24].

**Figure 2:**
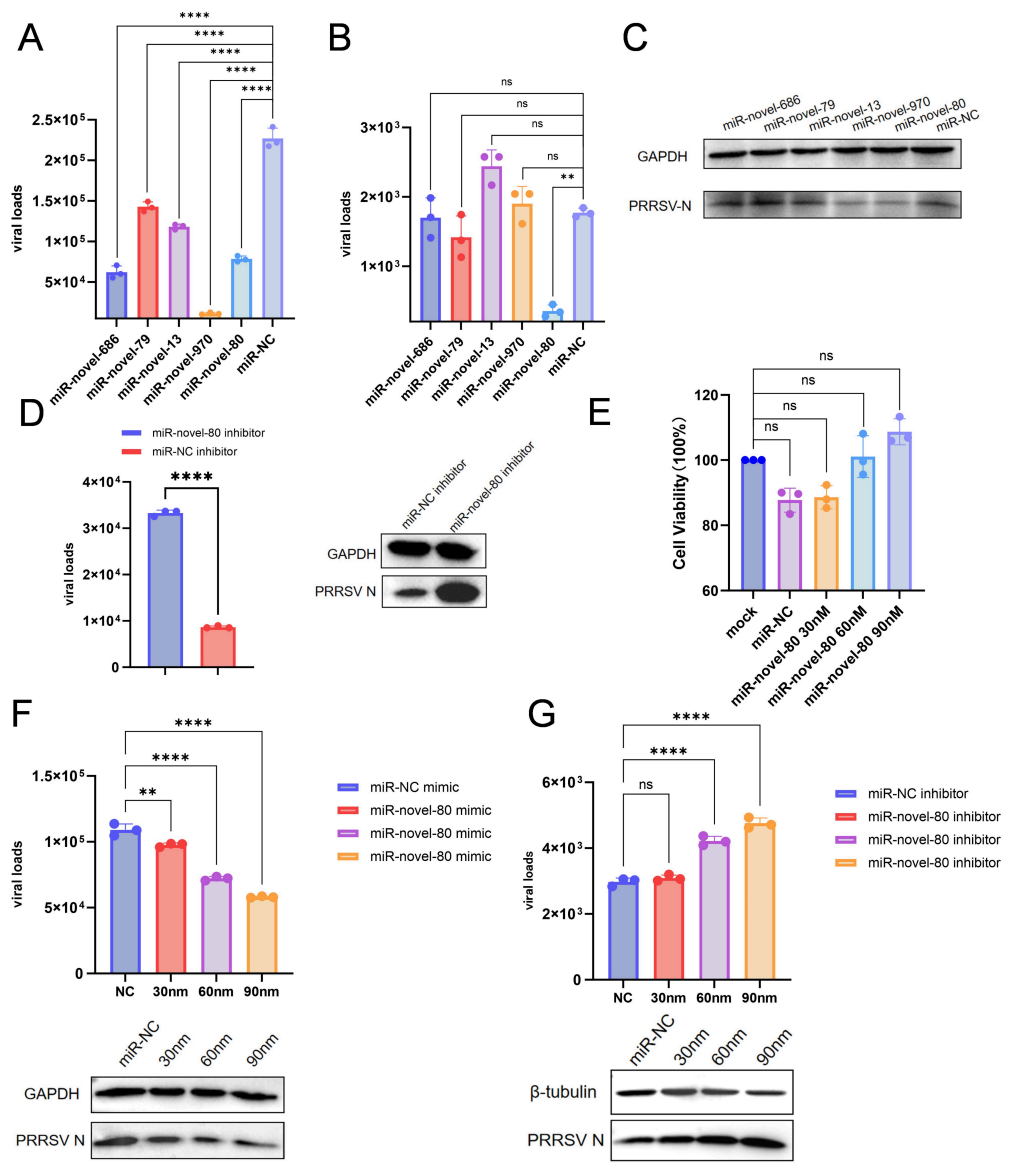
miR-novel-80 inhibits PRRSV replication. (**A**,**B**) MARC-145 cells (**A**) and iPAMs (**B**) were transfected with the indicated miRNA mimics or negative control (NC) mimics for 10 h, followed by infection with the PRRSV JXA1 strain (MOI = 0.1) for 24 h. Viral copy numbers were subsequently determined via RT-qPCR. ** *p* < 0.01, **** *p* < 0.0001, ns: no significantly. (**C**) iPAMs were transfected and infected under the same conditions as in (**A**,**B**), and viral protein levels were assessed by WB analysis. (**D**) iPAMs were transfected with miR-novel-80 inhibitors or NC inhibitors for 10 h, followed by infection with PRRSV JXA1 (MOI = 0.1) for 24 h. PRRSV replication was evaluated using both RT-qPCR and Western blotting analyses. **** *p* < 0.0001. (**E**) A CCK-8 assay was performed on iPAMs transfected with 30, 60, or 90 nM of miR-novel-80 mimics or NC for 24 h. ns: no significantly. (**F**,**G**) iPAMs were transfected with graded doses of miR-novel-80 mimics (**F**) or inhibitors (**G**), followed by PRRSV infection for 24 h. Viral replication was subsequently analyzed by RT-qPCR and WB. ** *p* < 0.01, **** *p* < 0.0001, ns: no significantly.

Currently, the roles of novel miRNAs in PRRSV infection remain largely unexplored. To prioritize candidates for functional validation, we focused on the 32 novel miRNAs identified from the TP challenge vs. LW challenge comparison, as our primary aim was to identify miRNAs associated with breed-specific susceptibility to PRRSV following infection. From these 32 novel miRNAs, five candidates with relatively high statistical significance were selected for initial functional validation, including miR-novel-686 (*p* < 0.0001), miR-novel-79 (*p* < 0.001), miR-novel-13 (*p* < 0.001), miR-novel-80 (*p* < 0.001), and miR-novel-970 (*p* < 0.05) (Table S1). To evaluate their impact on viral replication in vitro, Marc-145 cells were transfected with the respective miRNA mimics or negative control (NC) mimics for 10 h, followed by infection with JXA1-like PRRSV (MOI = 0.1), and the cells were harvested at 24 h post-infection (hpi). Compared with the NC mimics, all five miRNA mimics significantly reduced the viral copy number, indicating a highly significant reduction (*p* < 0.001) (Figure 2A). The data verified that only miR-novel-80 significantly reduced the viral load of PRRSV in iPAMs (Figure 2B). Consistently, WB analysis demonstrated that the PRRSV N protein level was markedly reduced upon treatment with miR-novel-80 mimics (Figure 2C). In contrast, the miR-novel-80 inhibitor significantly (*p* < 0.0001) facilitated PRRSV replication in iPAMs. Moreover, the miR-novel-80 inhibitor also increased the expression of the PRRSV N protein, as shown by WB analysis (Figure 2D). Additionally, we assessed the cytotoxic effects of the miRNA mimics in iPAMs at concentrations of 30, 60, and 90 nM, and found that none of the tested concentrations exhibited any cytotoxicity in the cells (Figure 2E). Overexpression of miR-novel-80 inhibited PRRSV infection in a dose-dependent manner (Figure 2F). Conversely, the downregulation of miR-novel-80 promoted PRRSV infection in a dose-dependent manner (Figure 2G). Collectively, our results indicate that miR-novel-80 exerts antiviral activity against PRRSV.

### 3.2. miR-Novel-80 Directly Targets the PRRSV nsp1 Gene

**Figure 3:**
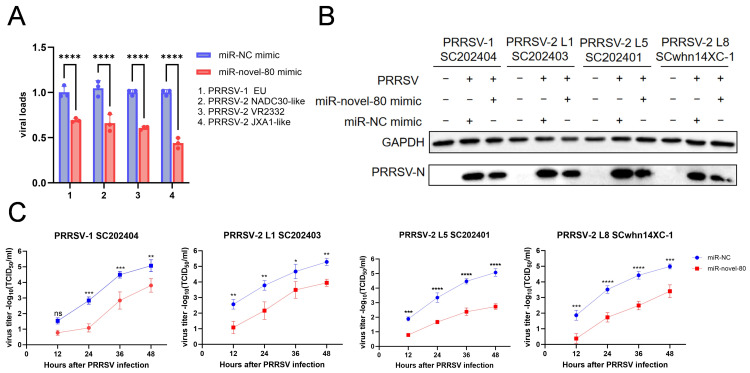
miR-novel-80 directly targets the PRRSV type region. (**A**,**B**) iPAMs were transfected with miR-novel-80 mimics or NC and then infected with the indicated PRRSV strains [PRRSV-2 strains: SC202403, SC202401, SCwhn14XC-1 and SC202404 at an MOI of 0.05. At 24 hpi, viral RNA levels (**A**) and N protein expression (**B**) were measured to assess viral replication. miR-novel-80 inhibits the replication of multiple PRRSV genotypes and lineages. **** *p* < 0.0001. (**C**) Viral titers determined by TCID_50_ assays at indicated time points post-transfection with miR-novel-80. * *p* < 0.05, ** *p* < 0.01, *** *p* < 0.001, **** *p* < 0.0001, ns: no significantly.

To examine whether the inhibitory effect of miR-novel-80 on PRRSV replication is strain-dependent, we infected miR-novel-80-transfected iPAMs with various PRRSV strains, including three PRRSV-2 strains [SC202403 (NADC30-like, L1), SC202401 (VR2332-like, L5), and SCwhn14XC-1 (JXA1-like, L8)] and one PRRSV-1 strain [SC202404 (BJEU06-1-like, L1)] (MOI = 0.05). The results demonstrated that miR-novel-80 effectively inhibited the replication of multiple PRRSV genotypes and lineages at both the viral RNA level (Figure 3A) and N protein expression level (Figure 3B). Subsequently, a TCID_50_ assay was applied to monitor the dynamics of PRRSV replication in miR-novel-80-overexpressing iPAMs. The data showed that viral growth was significantly repressed compared to the NC group at 24–48 hpi (Figure 3C).

**Figure 4:**
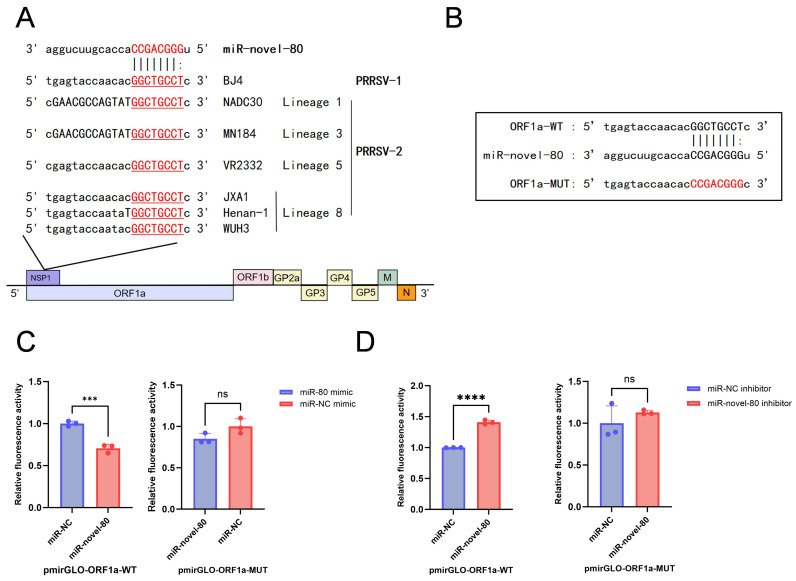
miR-novel-80 directly targets the conserved *nsp1* gene of PRRSV. (**A**) Sequence alignment of the miR-novel-80 target site within the nsp1-coding region (ORF1a) of PRRSV. The target site was predicted using miRanda (v3.3a) and is highly conserved across PRRSV-1 and PRRSV-2 lineages (L1, L3, L5, and L8). (**B**) Schematic diagram of the predicted miR-novel-80 binding site and the corresponding mutant sequence within the PRRSV JXA1 genome. The wild-type (WT) or mutant (MUT) binding sites were cloned into the pmirGLO vector for dual-luciferase reporter assays. (**C**) Relative luciferase activity in 293T cells co-transfected with the dual-luciferase reporter plasmids and miR-novel-80 mimics or NC mimics for 24 h. *** *p* < 0.001, ns: no significantly. (**D**) Relative luciferase activity in 293T cells co-transfected with the dual-luciferase reporter plasmids and miR-novel-80 inhibitors or NC inhibitors for 24 h. **** *p* < 0.0001, ns: no significantly.

Bioinformatic predictions indicated that miR-novel-80 directly targets the *nsp1* gene within the ORF1a region of both PRRSV-1 and four lineages of PRRSV-2. To evaluate the evolutionary conservation of this target site, we aligned the sequences across multiple PRRSV genotypes/lineages. The miR-novel-80 binding site exhibits high conservation, displaying sequence identity across all analyzed PRRSV-2 lineages (L1, L3, L5, and L8) and the representative PRRSV-1 strain. Specifically, this target region is mapped to nucleotides 962–969 in L1 (NADC30-like), L3 (MN184), and L5 (VR2332) strains, and to nucleotides 961–968 in L8 strains (JXA1-like, WUH3, and Henan) alongside the PRRSV-1 strain BJ4 (Figure 4A). To verify whether miR-novel-80 directly targets the predicted binding site within the PRRSV genome, we constructed a wild-type reporter plasmid (pmirGLO-ORF1a-WT) containing the putative binding site and a mutant plasmid (pmirGLO-ORF1a-MUT) with a mutated binding site (Figure 4B). These dual-luciferase reporter plasmids were co-transfected with miR-novel-80 mimics into 293T cells. The luciferase reporter assay results showed that miR-novel-80 mimics significantly (*p* < 0.001) reduced the luciferase activity in cells transfected with pmirGLO-ORF1a-WT, whereas the luciferase activity of pmirGLO-ORF1a-MUT was not affected (Figure 4C). Conversely, treatment with the miR-novel-80 inhibitor significantly (*p* < 0.0001) increased luciferase activity in cells transfected with pmirGLO-ORF1a-WT but had no effect on those transfected with pmirGLO-ORF1a-MUT (Figure 4D).

### 3.3. Transcriptomic Analysis of miR-Novel-80 in iPAMs

**Figure 5:**
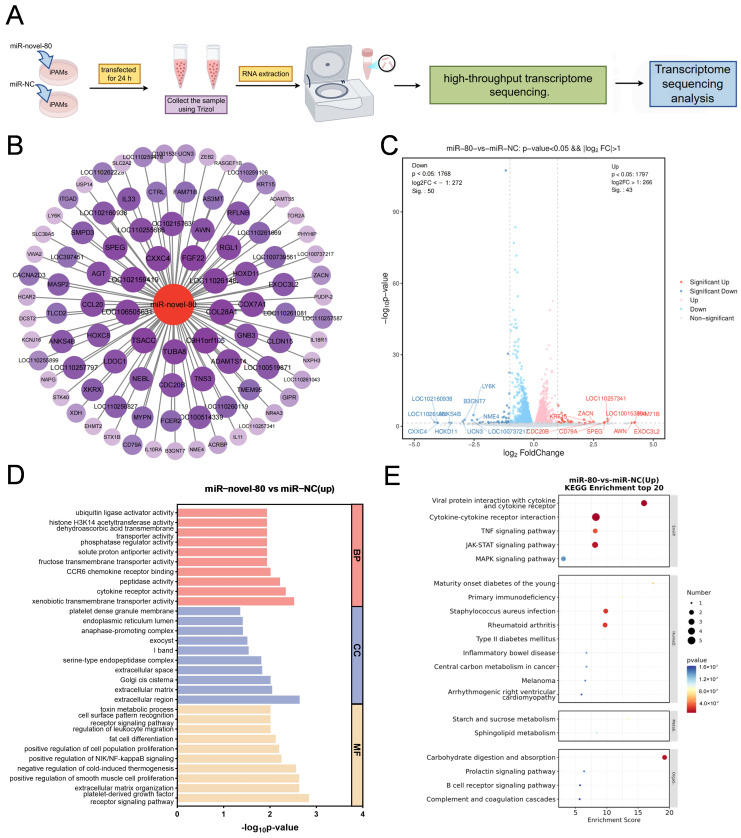
Transcriptomic analysis of miR-novel-80 in iPAMs. (**A**) Schematic overview of the experimental procedure: iPAMs were transfected with miR-novel-80 mimics or NC mimics for 24 h. Total RNA was then extracted using the TRIzol reagent, followed by high-throughput transcriptome sequencing. (**B**) Network visualization of potential miR-novel-80 target genes. Node size and color intensity correspond to the magnitude of differential expression. (**C**) Volcano plot showing the distribution of DEGs between the miR-NC and miR-novel-80 groups. The vertical dashed lines indicate the fold-change threshold (|log_2_FC| > 1), and the horizontal dashed line indicates the significance threshold (*p* < 0.05). (**D**) Gene ontology (GO) enrichment analysis of the identified DEGs, categorized by biological process (BP), cellular component (CC), and molecular function (MF). (**E**) KEGG pathway enrichment analysis of the DEGs.

Given the antiviral activity of miR-novel-80, we performed high-throughput transcriptomic analysis to elucidate the underlying host biological processes modulated by this novel miRNA. iPAMs were transfected with 60 nM of either miR-novel-80 mimics or NC mimics for 24 h. Each experimental condition was performed in three biological replicates. Total RNA was extracted using the TRIzol reagent and subjected to transcriptomic profiling (Figure 5A). Analysis of the transcriptome sequencing data identified 93 potential target genes associated with miR-novel-80 (Figure 5B), comprising 43 upregulated and 50 downregulated genes. A volcano plot was used to visualize the distribution of these differentially expressed genes (DEGs) between the miR-NC and miR-novel-80 groups (Figure 5C). Transcriptomic analysis revealed several significant DEGs, such as *CXXC4*, *LY6K*, and *COX7A1*. Gene ontology (GO) enrichment analysis indicated that the upregulated genes were significantly enriched in biological processes, such as pattern recognition receptor signaling, leukocyte migration, and the positive regulation of the NIK/NF-κB signaling pathway (Figure 5D). Furthermore, KEGG pathway analysis revealed that these upregulated genes were primarily involved in immune-related pathways, such as “viral protein interaction with cytokine and cytokine receptor” (fold enrichment = 15.0) and “cytokine-cytokine receptor interaction” (fold enrichment = 12.5) (Figure 5E). Because these DEGs were significantly enriched in pattern recognition receptor signaling, leukocyte migration, and the NIK/NF-κB signaling pathway, we speculate that miR-novel-80 contributes to the enhancement of the host immune response against PRRSV.

### 3.4. miR-Novel-80 Targets the Host CXXC4

**Figure 6:**
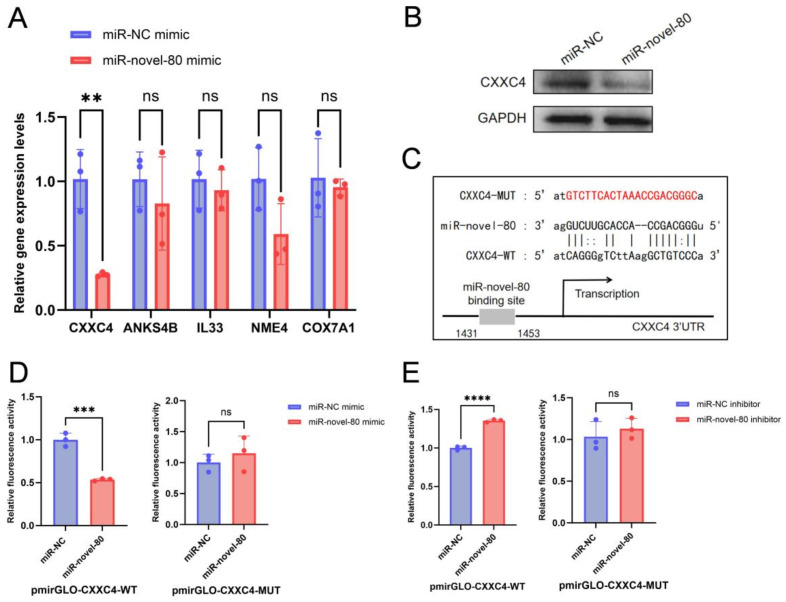
miR-novel-80 directly targets host *CXXC4*. (**A**) RT-qPCR analysis of *CXXC4*, *ANKS4B*, *Il33*, *NME4*, and *COX7A1* mRNA levels in iPAMs transfected with miR-novel-80 mimics for 24 h. ** *p* < 0.01, ns: no significantly. (**B**) WB analysis of CXXC4 protein expression in iPAMs at 24 h post-transfection with miR-novel-80 mimics. (**C**) Schematic representation of the predicted miR-novel-80 binding site and the corresponding mutant sequence within the 3′UTR of the *CXXC4* gene. (**D**,**E**) Dual-luciferase reporter assays in 293T cells co-transfected with pmirGLO-CXXC4-WT or pmirGLO-CXXC4-MUT reporter plasmids alongside miR-novel-80 mimics (**D**) or miR-novel-80 inhibitors (**E**) for 24 h. *** *p* < 0.001, **** *p* < 0.0001, ns: no significantly.

To identify potential target host genes, we analyzed the transcriptome sequencing data and selected five candidate genes, namely *CXXC4*, *ANKS4B*, *Il33*, *NME4*, and *COX7A1*, that might be regulated by miR-novel-80. iPAMs were transfected with miR-novel-80 mimics for 24 h, after which the transcriptional levels of the candidate genes were quantified by RT-qPCR. The results showed that the mRNA levels of *CXXC4* were significantly downregulated (*p* < 0.01) by miR-novel-80, whereas *ANKS4B*, *IL33*, *NME4*, and *COX7A1* showed no significant changes (Figure 6A). Consistent with the mRNA results, CXXC4 protein expression levels were also significantly decreased following miR-novel-80 transfection compared with the NC group (Figure 6B). Bioinformatic analysis with miRanda indicated that miR-novel-80 targets the 3′UTR of *CXXC4*. To confirm whether miR-novel-80 directly targets the 3′UTR of *CXXC4*, dual-luciferase reporter plasmids carrying the *CXXC4* 3′UTR with the wild-type or base-pair mutant miR-novel-80 binding regions were constructed (Figure 6C). Dual-luciferase reporter assays showed that miR-novel-80 significantly (*p* < 0.001) suppressed the luciferase activity of cells transfected with pmirGLO-CXXC4-WT, whereas it had no effect on the activity of pmirGLO-CXXC4-MUT (Figure 6D). Conversely, the miR-novel-80 inhibitor significantly (*p* < 0.0001) enhanced the luciferase activity of cells transfected with pmirGLO-CXXC4-WT but had no significant effect on the activity of pmirGLO-CXXC4-MUT (Figure 6E).

### 3.5. CXXC4 Promote PRRSV Replication

**Figure 7:**
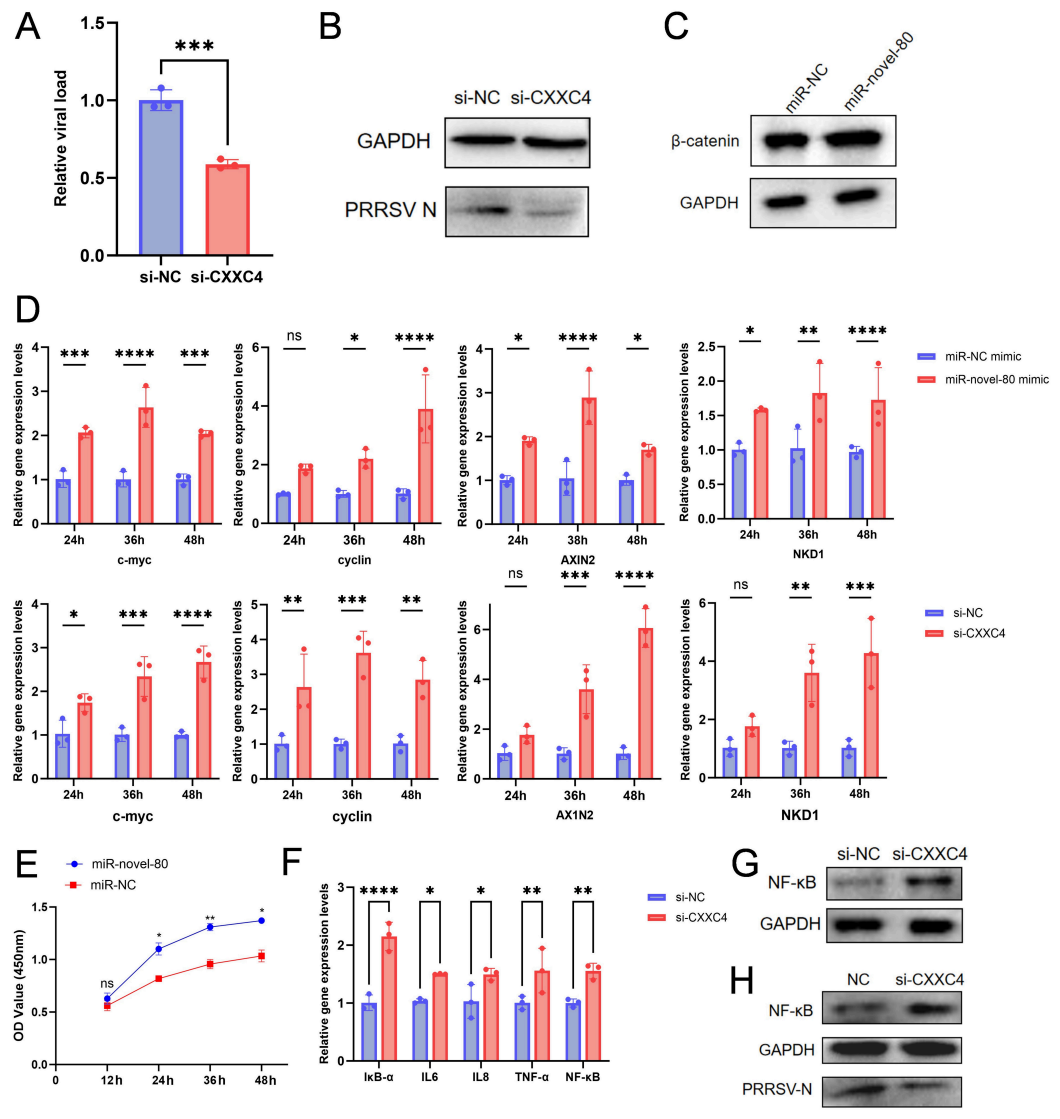
miR-novel-80 inhibits PRRSV replication by targeting host CXXC4 to activate Wnt/β-catenin and NF-κB signaling. (**A**,**B**) iPAMs at 70% confluence were transfected with si-CXXC4, followed by PRRSV inoculation. PRRSV replication was assessed by RT-qPCR (**A**) and WB (**B**). *** *p* < 0.001. (**C**) WB analysis of β-catenin protein levels in iPAMs transiently transfected with miR-novel-80 mimics for 24 h. (**D**) RT-qPCR analysis of Wnt/β-catenin target genes (*AXIN2*, *NKD1*, *c-Myc*, and *cyclin D1*) in iPAMs transfected with miR-novel-80 mimics, si-CXXC4, or their respective negative controls at 24, 36, and 48 h post-transfection. * *p* < 0.05, ** *p* < 0.01, *** *p* < 0.001, **** *p* < 0.0001, ns: no significantly. (**E**) Cell viability was determined using the CCK-8 assay at the indicated time points after transfection with NC or miR-novel-80 mimics. * *p* < 0.05, ** *p* < 0.01, ns: no significantly. (**F**) RT-qPCR analysis of *IκB-α*, *IL-6*, *IL-8*, *TNF-α*, and *NF-κB* mRNA levels in iPAMs transfected with si-CXXC4 for 48 h. * *p* < 0.05, ** *p* < 0.01, **** *p* < 0.0001. (**G**) WB analysis of NF-κB p65 protein levels in iPAMs transfected with si-CXXC4 for 48 h. (**H**) Western blot analysis of NF-κB p65 and PRRSV N protein levels in iPAMs transfected with si-CXXC4 or si-NC for 48 h, followed by PRRSV infection for 24 h.

To further verify the role of *CXXC4* in PRRSV infection, iPAMs were transfected with si-CXXC4 at 70% confluence, followed by PRRSV inoculation. The results showed that si-CXXC4 significantly (*p* < 0.01) inhibited PRRSV replication at the mRNA level (Figure 7A). Furthermore, WB analysis demonstrated that the knockdown of CXXC4 markedly reduced PRRSV N protein levels compared with the NC group (Figure 7B). These results suggested that CXXC4 facilitates PRRSV replication.

### 3.6. miR-Novel-80 Inhibits PRRSV Replication by Downregulating CXXC4

Previous reports showed *CXXC4* as a negative regulator of the Wnt/β-catenin signaling pathway [21,22]. To investigate whether miR-novel-80 activates this pathway via CXXC4 downregulation, we transfected iPAMs with miR-novel-80 mimics for 24 h. WB analysis showed that the protein levels of β-catenin were significantly upregulated (Figure 7C). To validate the activation of the Wnt/β-catenin signaling pathway, we quantified the mRNA expression levels of its responsive genes (*AXIN2*, *NKD1*, *c-Myc*, and *cyclin D1*) in iPAMs following transfection. We transfected iPAMs with miR-novel-80 mimics or si-CXXC4 for different times. The mRNA levels of these four candidate genes were consistently regulated at 24, 36, and 48 h post-transfection (Figure 7D). Functional assays further demonstrated that miR-novel-80-mediated *CXXC4* suppression activates the Wnt/β-catenin pathway, we assessed iPAM proliferation following miR-novel-80 transfection. CCK-8 assays revealed that miR-novel-80 significantly promoted cell proliferation compared with the NC group at 24, 36, and 48 h (Figure 7E). The Wnt/β-catenin signaling pathway has been shown to restrict virus replication by enhancing the NF-κB-dependent innate immune response [25]. We further examined the expression of multiple NF-κB-related inflammatory factors and found that knockdown of *CXXC4* significantly upregulated the mRNA levels of *IκBα* (*p* < 0.0001), *Il6* (*p* < 0.05), *Il8* (*p* < 0.05), *Tnfα* (*p* < 0.01), and *NfκB* (*p* < 0.01) (Figure 7F). Furthermore, Western blot analysis revealed that *CXXC4* knockdown significantly increased the protein expression levels of NF-κB (Figure 7G). To determine whether *CXXC4* knockdown inhibits PRRSV replication via NF-κB activation, iPAMs were transfected with si-CXXC4 for 48 h and subsequently infected with PRRSV for 24 h. WB analysis revealed that *CXXC4* knockdown enhanced NF-κB protein expression and suppressed PRRSV N protein levels compared with the control (Figure 7H). These results revealed that miR-novel-80 relieves repression of the Wnt/β-catenin signaling pathway by targeting *CXXC4*, thereby activating NF-κB and suppressing PRRSV replication.

## 4. Discussion

PRRS has been endemic globally for over three decades, posing a persistent threat to the commercial swine industry. The pathogenesis of PRRSV, characterized by rapid mutation rates and sophisticated immune evasion strategies driven by immune selection, renders conventional prophylactic measures largely insufficient [2,26]. Therefore, it is particularly important to elucidate the regulatory mechanisms of virus–host interactions. Operating as crucial post-transcriptional regulators, miRNAs govern intracellular protein levels by altering mRNA stability and translational efficiency. Beyond their physiological functions, accumulating evidence underscores the dual capability of miRNAs to influence RNA virus replication and pathogenesis [27,28]. Specifically, host miRNAs have been shown to employ diverse mechanisms to regulate PRRSV infection [29]. In the current study, we found that miR-novel-80 was differentially expressed in PAMs collected from Tibetan pigs and Large White pigs following a PRRSV challenge. Furthermore, the overexpression of miR-novel-80 inhibited, in a dose-dependent manner, the replication of a PRRSV-1 strain and multiple lineages (L1, L5, and L8) of PRRSV-2 strains. Mechanistically, we demonstrated that this antiviral activity is attributable to both the direct targeting of the PRRSV *nsp1*-coding region within the ORF1a region and the indirect activation of the Wnt/β-catenin signaling pathway via the suppression of host *CXXC4*. Collectively, this study provides the first biological characterization of miR-novel-80 and unveils its underlying molecular mechanisms in restricting PRRSV infection.

Multiple lines of evidence showed that host miRNAs could bind to a broad range of RNA viruses, directly regulating their pathogenesis. There have been many reports showing that well-known miRNAs, such as miR-130b, miR-320, miR-23, miR-150, miR-181, and the miR-let-7 family [19,20,30,31,32,33], can directly interact with PRRSV and subsequently inhibit viral replication. However, in this study, a large number of novel miRNAs were differentially expressed in Tibetan pigs compared with Large White pigs. To investigate the effects of these novel transcripts on PRRSV replication, five candidate miRNAs (miR-novel-686, -79, -13, -80, and -970) were selected. Interestingly, our results demonstrated that miR-novel-80 effectively suppressed PRRSV infection in both MARC-145 cells and iPAMs, further confirming that host cellular miRNAs serve as crucial defense mechanisms against PRRSV. More importantly, we validated the antiviral spectrum of miR-novel-80 across distinct PRRSV strains, revealing a significant suppression of viral replication regardless of the genotype/lineage. Previous studies indicated that multiple miRNAs exclusively inhibit PRRSV-2 strains but fail to antagonize PRRSV-1 viruses [19]; Notably, to the best of our knowledge, miR-novel-80 is the first miRNA shown to effectively inhibit both PRRSV-2 and PRRSV-1 genotypes [19,34,35], filling a critical gap in the current understanding of miRNA-mediated cross-genotypic antiviral activity. Therefore, miR-novel-80 represents a potential biomarker and genetic target for understanding viral infection restriction and informing the selection of PRRSV-resistant pigs.

Recent evidence demonstrates that miRNAs can also modulate PRRSV replication and pathogenesis through indirect virus-mediated changes in the host transcriptome [35,36]. The regulatory roles of miRNAs in PRRSV infection are now relatively well understood. miR-181 and miR-124a target the 3′UTR of PRRSV receptor CD163, consequently reducing CD163 expression and ultimately inhibiting viral replication [33,37]. Similarly, other PRRSV entry receptors, including CD151 and MYH9, can be downregulated by miR-506 and miRNA-let-7f-5p, respectively [20,38]. Elucidation of the miRNA-mediated regulation of PRRSV entry advances our understanding of PRRSV cell tropism. In addition to modulating viral entry, miRNAs also critically regulate molecules associated with the host’s anti-PRRSV immune response. For example, PRRSV-induced upregulation of miR-373 represses type I IFN production, thereby promoting viral replication [39]. In contrast, miR-125b fine-tunes the host response by modulating NF-κB activation [40]. Moreover, the miR-let-7 family influences downstream inflammatory cytokine pathways [20]. Consistent with this immune-modulating paradigm, our investigation into miR-novel-80 revealed a profound capability to orchestrate host antiviral responses. High-throughput transcriptome sequencing of cells transfected with miR-novel-80 exhibited a significant upregulation in pattern recognition receptor (PRR) signaling, leukocyte migration, and the NIK/NF-κB signaling pathway. To elucidate the molecular basis driving this robust immune activation, we sought to identify the direct downstream targets of miR-novel-80. Our findings established that miR-novel-80 directly targets and downregulates the expression of *CXXC4*, a well-documented negative regulator of the Wnt/β-catenin signaling pathway [21,22]. Consequently, the miR-novel-80-mediated suppression of *CXXC4* effectively relieves this inhibition, culminating in the activation of the Wnt/β-catenin cascade.

The Wnt/β-catenin axis is widely recognized for its complex and pivotal roles in host immune modulation during viral infections [41,42]. Indeed, Hao et al., demonstrated that pharmacological activation of this pathway using lithium chloride (LiCl) effectively antagonizes PRRSV infection and profoundly shapes inflammatory responses [43]. Integrating our transcriptomic data with these mechanistic insights, we propose a coherent antiviral model: miR-novel-80 triggers the Wnt/β-catenin signaling cascade via the targeted degradation of *CXXC4*; this Wnt activation, in turn, drives the NIK/NF-κB innate immune axis and PRR signaling, thereby mounting a formidable antiviral defense that ultimately restricts PRRSV replication. Collectively, these findings outline a mechanistic framework by which miR-novel-80 modulates host-directed immune responses through the CXXC4/Wnt/β-catenin/NF-κB signaling axis to restrict PRRSV replication in vitro.

Nevertheless, the clinical translation of miRNA-based approaches must account for the inherent complexity and potential non-specificity of miRNA regulatory networks. Genome-wide analyses indicate that over 60% of protein-coding genes contain conserved miRNA targets [44]. Meaning a single miRNA can regulate hundreds of downstream messages. Conversely, a single mRNA (such as *CXXC4*) is often subject to combinatorial regulation by multiple endogenous miRNAs, a functional redundancy evidenced by the fact that many miRNAs are dispensable for baseline organismal viability [45]. This broad-spectrum regulatory nature frequently triggers off-target effects and systemic toxicities, as starkly highlighted by the discontinuation of the MRX34 clinical trial [46]. Therefore, although our in vitro data demonstrates that miR-novel-80 potently restricts PRRSV replication via the Nsp1/Wnt axis, the possibility of systemic toxicities and off-target effects cannot be completely excluded. Given these challenges, further validation in PRRSV-infected pig models is essential. To rigorously assess the in vivo efficacy and safety profile of miR-novel-80, future studies incorporating comprehensive histopathology and immunohistochemistry will be critical, providing a more robust foundation for its potential therapeutic application.

Currently, the prophylactic landscape against PRRSV is predominantly vaccine driven [7,9]. However, inherent limitations, such as insufficient cross-protection and vaccine-strain recombination, underscore a critical demand for alternative interventions [47,48,49]. In this study, our results demonstrate the importance of the miR-novel-80 in modulating PRRSV replication in vitro. However, translating the in vitro potency of miR-novel-80 into meaningful in vivo outcomes faces extensive bottlenecks. As corroborated by current clinical literature, the broader translation of RNA-based therapeutics remains severely restricted by systemic vulnerabilities, such as transient stability, poor target-tissue selectivity, and severe immunogenicity [50,51]. Given these substantial translational barriers, the current value of our findings lies primarily in advancing the mechanistic understanding of host–virus interactions. Future studies are therefore warranted to further validate the functional relevance of miR-novel-80 in vivo and to fully delineate its regulatory network.

## 5. Conclusions

In conclusion, a novel miRNA, miR-novel-80, was identified from PRRSV-challenged Tibetan pigs versus Large White pigs. Over-expression of miR-novel-80 inhibited the replication of the PRRSV-1 strain and multiple lineages of PRRSV-2 isolates in a dose-dependent manner. The antiviral activity of miR-novel-80 was attributable to the direct targeting of the PRRSV *nsp1* gene and indirect activated the Wnt/β-catenin signaling pathway via inhibiting the expression of host *CXXC4.* Overall, our study identifies miR-novel-80 as a novel regulator of PRRSV replication, acting through both direct and indirect mechanisms, and provides mechanistic insights into miRNA-mediated antiviral defense with potential implications for understanding PRRSV-host interactions.

## Figures and Tables

**Table 1:** The miRNA sequences.

Name	Sequences (5′–3′)
ssc-miR-novel-686 mimic	ACAGGGCUGCUGGCAUCCACG
ssc-miR-novel-79 mimic	GCGGCUGGCGGCCUGGACGCCGUG
ssc-miR-novel-13 mimic	CGGGGUUUUGAGGGCGAGAUGA
ssc-miR-novel-80 mimic	UGGGCAGCCACCACGUUCUGGA
ssc-miR-novel-970 mimic	UGGCAGCCACCACGUUCUGGA
ssc-miR-novel-80 inhibitor	UGGGCAGCCACCACGUUCUGGA
miR-NC mimic	UCACAACCUCCUAGAAAGAGUAGA
miR-NC inhibitor	UCUACUCUUUCUAGGAGGUUGUGA
pig-siCXXC4	GCUGAAGCAGAGAUAAUG

**Table 2:** Primers for qRT-PCR.

Genes	Sequences (5′–3′)
PRRSV ORF7-F	AATAACAACGGCAAGCAGCA
PRRSV ORF7-R	GCACAGTATGATGCGTCGGC
CXXC4-F	GGGGCATCTCATTACCTCCA
CXXCE-R	GCAATTTGAAACGCACTGTCTG
ANKS4B-F	ATAATTTGCAGCAGAGGAGGGG
ANKS4B-R	CAGTGGCGGCCTTATCCAA
IL33-F	AAACCAGATCACAAGAAGCCTG
IL33-R	TATCGAGACTTTGCCGGCTG
NME4-F	CCCCAACTCGGGTTGTCCC
NME4-R	GAAGCCCCTCCTCTCAAAGC
COX7A1-F	CAGAAAATCTTCCAGGCGGAC
COX7A1-R	ACAGGCTATAGACAGTGCCC
CyclinD1-F	ATGCTGAAGGCGGAGGAGACC
CyclinD1-R	TCCAGGTGGCGACGATCTTCC
C-myc-F	CTGAGGAGGAACAAGAAGATGA
C-myc-R	TCCAGCAGAAGGTGATCCAGACTC
AXIN2-F	CTCAGTAACAGCCCGAGAGC
AXIN2-R	AGGCGAGTCACCAACATAGC
NKD1-F	ATCGAGGAGTGGATCGGGAG
NKD1-R	ACATCGCACTGGAGCTCTTC
IκB-α-F	TGCTGAGGTGGGTGTCATTG
IκB-α-R	CCATCAAGTCTCCCTGACGC
IL6-F	CAGCCCTGAGAAAGGAGACA
IL6-R	CCAGGCAAGTCTCCTCATTG
IL8-F	AGGACAAGAGCCAGGAAG
IL8-R	CTGCACCTTCACACAGAGC
TNF-α-F	TCTGTCTGCTGCACTTTGGAGTGA
TNF-α-R	TTGAGGGTTTGCTACAACATGGGC
NF-κB-F	CTCGCACAAGGAGACATGAA
NF-κB-R	ACTCAGCCGGAAGGCATTAT
GAPDH-F	GTCGGTTGTGGATCTGACCT
GAPDH-R	AGCTTGACGAAGTGGTCGTT

## Data Availability

The original contributions presented in this study are included in the article/Supplementary Material. Further inquiries can be directed to the corresponding author.

## Supplementary Materials

The following supporting information can be downloaded at https://www.mdpi.com/article/10.3390/ani16152434/s1: Table S1: Differentially expressed novel miRNAs in TP challenge vs. LW challenge.
