# Supplementary material for: miR-Novel-80 Suppresses Porcine Reproductive and Respiratory Syndrome Virus Replication by Targeting the Viral Nsp1 Gene and Downregulating Host CXXC Finger Protein 4
Source: Animals (Basel). 2026 Aug 6;16(15):2434. doi: 10.3390/ani16152434 (PMC13464968; doi:10.3390/ani16152434)
Supplement: Supplementary file 1 [file animals-16-02434-s001.zip › animals-4428992-supplementary.pdf]

Table S1. Differentially expressed novel miRNAs in TP challenge vs. LW challenge

miRNA_id	BaseMean_DESeq2_unpaired	FoldChange	log2FoldChange	p-value	q-value
novel686_mature	22.268880871532307	288.089652273563	8.17037403187016	0.000000049254905	3.0462264141688307e-06
novel13_mature	65.4426862313223	6.608639876278911	2.724353381315689	0.000111297047268451	0.00255665217153814
novel13_star	11.8821033822176	37.3998994249283	5.22496248533845	0.000471093049325025	0.008487008780758991
novel489_mature>novel490_mature	11.8152821022805	0.0281626162910051	-5.150075	0.00072348269928322	0.0118710222494634
novel79_mature	8.24686507310498	53.713138048406286	5.74720310449522	0.000838017185056802	0.0134753163357134
novel80_mature	11.847704164737198	0.0192306604399001	-5.700448	0.000883261676001175	0.0136565843750951
novel914_mature	8.207956012976931	0.0159090519921549	-5.974008	0.0009256401373075847	0.0140180367344236
novel27_mature>novel473_mature	128.973257369264	4.38613688386348	2.1329508367697	0.00144551699092349	0.0211308301945906
novel268_mature>novel697_mature	10.0862772835311	0.0365160222325907	-4.775327	0.00310079573142781	0.0402103188398057
novel46_mature	52.447803302595204	0.18761591918999893	-2.414146	0.00330826170899745	0.0421362723447333
novel58_mature	344.256626369591	0.3094088284908089	-1.692414	0.00537783425643009	0.0617682677452828
novel466_mature	11.893797640432195	13.8230418380898	3.789003218706221	0.00664489804082614	0.0752464510538622
novel1_mature>novel810_mature	6098.4476132301415	0.37317445480981	-1.422078	0.00883646428899168	0.0922664582902508
novel954_mature	11.0323735083696	0.0691475669802988	-3.854178	0.01205132537649	0.116738139791541
novel814_mature>novel955_mature	5.83357828269239	0.0370139391299951	-4.755788	0.0125194482075527	0.118419251280851
novel5_star	5437.8330959327295	0.3777114368462701	-1.404644	0.0130304175181298	0.12181925214623701
novel43_mature	6.4827357996217	0.0458595829337218	-4.446633	0.0152560389392562	0.13628728119068906
novel48_mature	5.69953116178113	0.0455140505673744	-4.457544	0.0186800458766051	0.15170461499788399
novel296_mature	4.27840577138386	0.0312587748292273	-4.999595	0.0209544482020295	0.162635555589455
novel895_mature>novel952_mature	18.7219172854718	0.151193159370612	-2.725535	0.0216442841393927	0.162635555589455

miRNA_id	BaseMean_DESeq2_unpaired	FoldChange	log2FoldChange	p-value	q-value
novel373_mature>novel413_mature	6.77709279750524	12.7284617280474	3.66998617073197	0.0271309839912003	0.193038151583408
novel7_mature>novel970_mature	6914.351748951701	0.487405488785061	-1.036806	0.030282782440516007	0.20633353459470202
novel383_mature	7.8029958472245005	0.0897496721453748	-3.47795	0.0335409714096081	0.221040500109221
novel29_mature	34.4908759052154	4.233229134543112	2.08175858039325	0.0349038263826538	0.22815184074515196
novel747_mature	3.707107710098912	0.0371414750351826	-4.750825	0.0371219961810999	0.24069423330326106
novel641_mature	4.09282714098261	19.9895884198272	4.32117686257835	0.040217805995244	0.256627904922033
novel202_mature	4.7795973997609	0.0653894747585557	-3.934798	0.0473482711735267	0.29059549636271303
novel30_mature	10.822671251089	4.6718084	2.2239811226056	0.0490043400288394	0.29820550679142105
novel5_mature	41.68659133491021	0.049841198258545	-4.326517	5.72508365008657e-06	0.000170480268691467
novel714_mature	71.9575714161823	0.0132773225415894	-6.234892	6.81846089213749e-08	3.6546950381857e-06
novel58_star	29.0220262938944	0.0845615688727148	-3.563854	8.33161052685499e-05	0.0021608435043843303
novel259_star	30.1463454776749	0.0454710179811602	-4.458909	9.14226684472758e-06	0.000253461467005551
